# The Emerging Role of Ferroptosis in Liver Cancers

**DOI:** 10.3390/life12122128

**Published:** 2022-12-16

**Authors:** Arianna Casini, Stefano Leone, Rosa Vaccaro, Giorgio Vivacqua, Ludovica Ceci, Luigi Pannarale, Antonio Franchitto, Paolo Onori, Eugenio Gaudio, Romina Mancinelli

**Affiliations:** 1 Department of Anatomical, Histological, Forensic Medicine and Orthopedic Sciences, Sapienza University of Rome, 00161 Rome, Italy; 2 Integrated Research Center (PRAAB), Campus Biomedico University of Roma, Via Alvaro del Portillo 21, 00125 Rome, Italy; 3 Division of Gastroenterology and Hepatology, Department of Medicine, Indiana University School of Medicine, Indianapolis, IN 46202, USA; 4 Department of Movement, Human and Health Sciences, Division of Health Sciences, University of Rome ‘Foro Italico’, 00135 Rome, Italy

**Keywords:** ferroptosis, iron metabolism, liver cancer, hepatocarcinoma, cholangiocarcinoma

## Abstract

Liver cancer represents a global health challenge with worldwide growth. Hepatocellular carcinoma (HCC) is the most common type of liver cancer. Indeed, approximately 90% of HCC cases have a low survival rate. Moreover, cholangiocarcinoma (CC) is another malignant solid tumor originating from cholangiocytes, the epithelial cells of the biliary system. It is the second-most common primary liver tumor, with an increasing course in morbidity and mortality. Tumor cells always show high metabolic levels, antioxidant modifications, and an increased iron uptake to maintain unlimited growth. In recent years, alterations in iron metabolism have been shown to play an important role in the pathogenesis of HCC. Several findings show that a diet rich in iron can enhance HCC risk. Hence, elevated iron concentration inside the cell may promote the development of HCC. Growing evidence sustains that activating ferroptosis may potentially block the proliferation of HCC cells. Even in CC, it has been shown that ferroptosis plays a crucial role in the treatment of tumors. Several data confirmed the inhibitory effect in cell growth of photodynamic therapy (PDT) that can induce reactive oxygen species (ROS) in CC, leading to an increase in malondialdehyde (MDA) and a decrease in intracellular glutathione (GSH). MDA and GSH depletion/modulation are crucial in inducing ferroptosis, suggesting that PDT may have the potential to induce this kind of cell death through these ways. A selective induction of programmed cell death in cancer cells is one of the main treatments for malignant tumors; thus, ferroptosis may represent a novel therapeutic strategy against HCC and CC.

## 1. Introduction—What Is Ferroptosis?

Cell death by apoptosis represents a natural and active energy-dependent mechanism that drives several biological processes, such as embryogenesis, neurogenesis and tissue renewal. It also defends organisms from pathogens and triggers anti-oncogenic signals inside cells. To date, the classification of the mechanisms of cell death by apoptosis has been enriched with several subtypes; therefore, there are many ways in which a cell can die [1]. According to the Death Nomenclature Committee in 2018, cell death is classified into accidental cell death (ACD) and regulated cell death (RCD) [2] depending on the activation process, which is either based or not based on a starting signal. While ACD depends on physical or mechanical cell damage leading to cell death, RCD can be modulated by molecular or pharmacological mechanisms. RCD or programmed cell death, in turn, can be classified as apoptotic or non-apoptotic death. There are many non-apoptotic-death-programmed mechanisms and include the following: (i) death dependent on vacuole formation (autophagy, entosis, methuosis and paraptosis); (ii) mitochondria involvement (mitoptosis and parthanatos); (iii) host immune response (pyroptosis and NETosis); and/or (iv) iron-dependent death (ferroptosis), which appears to be a non-apoptotic mechanism leading to neither chromatin fragmentation nor poly (ADP-ribose) polymerase (PARP) cleavage. Dixon SJ et al. demonstrated that the mechanism of iron-dependent death that can be reverted by iron chelators, but not by caspase inhibitors (e.g., Z-VAD-FMK), was first identified in 2012 [3]. The understanding of this death mechanism also has a growing interest due to numerous therapeutic implications. Studies suggest that cell models notoriously resistant to the pharmacological induction of apoptosis were vulnerable or sensitive to ferroptosis [4,5]. Given the altered turnover of cancer cells where cellular proliferation is predominant and unbalanced with respect to cell death, the induction, modulation or inhibition of ferroptosis through drugs or small molecules might be considered an innovative therapeutic strategy for the treatment of known drug-resistant tumors. Ferroptosis is a regulated cell death (RCD) mechanism and for this reason, it can be modulated and induced. From a biological point of view, it is a form of regulated cell death due to iron-dependent lipid peroxidation. Ferroptosis is triggered by a lack of activity of the enzyme glutathione peroxidase 4 (GPx4), which catalyzes the glutathione (GSH)-dependent reduction in membrane hydroperoxides to corresponding alcohols. In addition to the inactivation of GPx4, ferroptosis occurs when two other critical conditions are met: (i) the presence of aerobic metabolism, leading to the continuous formation of hydroperoxides starting from phospholipids; and (ii) the availability of reduced iron from the cellular iron pool. Morphologically, ferroptosis begins with cell contractions, which are characterized by the accumulation of perinuclear lipids and their subsequent diffusion into the cell cytoplasm. Indeed, lipid peroxidation can be completely blocked through either the depletion of iron or by acting directly on peroxidation itself or, more interestingly if we think about tumors, through the reduction in blood supply and the consequent decrease in oxygen availability. Several studies are progressively identifying molecules and small molecules capable of inducing ferroptosis or modulating it. Recent evidence has shown, for example, that the small molecule, RSL3, is inactivated by the protective enzyme GPx4 through an adapter molecule, 14-3-3ε [6]. Erastin was identified as a small molecule capable of selectively killing cells that overexpress the small T oncoprotein (ST) and the oncogenic rat sarcoma virus (RAS) protein. Subsequently, it was suggested to be a power inducer of ferroptosis [7]. To date, there are several hallmarks that identify ferroptosis, including the following: morphological features (cell swelling, plasma membrane rupture, smaller mitochondria, rupture of the outer mitochondrial layer); biochemical features (iron accumulation, greater lipid peroxidation and reduced activity of endogenous antioxidant molecules); protein abnormalities (upregulation of ACSL4 and acyl-CoA synthetase long-chain family member 4; TFRC, transferrin receptor; PTGS2, prostaglandin-endoperoxide synthase 2; CHAC1, ChaC glutathione specific gamma-glutamylcyclotransferase 1; degradation of ferritin, GPx4, ARNTL and aryl hydrocarbon receptor nuclear translocator-like; VDAC2/3, voltage-dependent anion channel 2/3); the release of damage-associated molecular patterns (DAMPs) (such as HMGB1 (high-mobility group box 1) and KRAS^G12D^, a mutated KRAS protein); and finally, genetic hallmarks (with the upregulation of PTGS2 and CHAC1 and the activation of NFE2L2 (nuclear factor erythroid 2-like 2)) [8]. The liver represents the organ that mainly coordinates the homeostasis of iron and holds its stores [9]. Furthermore, excessive lipid peroxidation is associated with a multiplicity of pathological conditions of the liver. The importance of ferroptosis in liver diseases, and in those that originate from the alteration of metabolism and liver iron homeostasis, is significantly increasing. A growing number of studies suggest that ferroptosis-induced cell death contributes to the pathogenesis of relevant liver diseases, such as hemochromatosis, alcohol-associated liver disease (ALD), HCV-infected viral hepatitis, liver fibrosis, NASH (non-alcoholic steatohepatitis), NAFLD (non-alcoholic fatty liver disease) and hepatobiliary carcinomas (HCCs and CCs) [10]. At the moment, a selective stain for ferroptotic cells in tissue sections is not available. However, Feng et al. found a specific antibody immunizing mice that react with membranes from lymphoma cells treated with piperazine erastin, a promoter of ferroptosis. In summary, they discovered an effective antibody (3F3 ferroptotic membrane antibody (3F3-FMA)). The antigen of 3F3-FMA is identified as the human transferrin receptor 1 protein for the accurate localization of ferroptosis through both immunofluorescence and flow cytometry applications [11].

## 2. Types of Liver Cancer: HCC and CC

**Figure 1:**
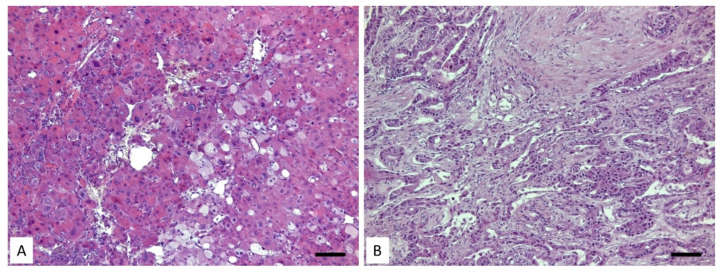
Representative H&E images of HCC (**A**) and CC (**B**) in which the abnormal morphology of liver tissue is evident. It is not possible to identify the typical cords of hepatocytes and the classic organization of the portal spaces (**A**,**B**). Scale bar: 50 μm.

Liver cancers represent the most common cause of cancer-related deaths. According to the World Health Organization, more than one million patients will die of liver cancer in 2030 [12]. HCC accounts for approximately 90% of cases. Some of the main risk factors are hepatitis B virus (HBV) and hepatitis C virus (HCV) infections, followed by other etiologies, such as cirrhosis, non-alcoholic steatohepatitis (NASH), metabolic syndrome and diabetes mellitus. All these factors increase the etiology of HCC [13,14,15]. The origin of the cancer is mainly linked to chronic liver disease with hepatic inflammation, fibrosis and irregular hepatocyte regeneration (Figure 1A), which may result in cirrhosis. Furthermore, these processes are characterized by genetic and epigenetic events that promote dysplastic nodule formation with proliferative, invasive and survival features leading to the completion of the transition to HCC [16]. It is possible to classify HCC in different molecular subtypes correlating with clinical features, namely the proliferation and the nonproliferation classes [17] (Table 1). The first one is present in patients with HBV infection, and it is characterized by an aggressive clinical profile that includes high serum levels of α-fetoprotein, poor cell differentiation and the activation of oncogenic pathways, such as the AKT–mammalian target of rapamycin (mTOR). In nonproliferative tumors class fall many cases that have mutations in the beta-catenin gene (CTNNB1) and that have a less aggressive prognosis [18]. Furthermore, another HCC classification based on cell origin and ontogeny has been provided, but it still debated. In fact, differences between tumors could be linked to the different types of cells residing in normal liver, from which different tumor subtypes could originate [19] (Table 1). Several murine models sustain the possibility that HCC arises from transformed mature hepatocytes, but other studies support that the source is liver stem cells, although common biochemical and histological features of HCC are detected in both types of origin [15]. Overall, these data highlight the concept that both tumor morphology and the epigenetic landscape of a tumor does not necessarily reflect its cell of origin [20]. Additional studies have proposed a molecular and immune classification [21]. The molecular classes are linked to specific genomic disorders and histopathological features. The proliferation class includes about 50% of HCCs with mutations in TP53 and amplifications of FGF19 or CCND1 [22]. Within the proliferation class, two subgroups can be identified: the proliferation of the progenitor cells or the uncontrolled proliferation of cells that activate the Wnt or TGFβ pathways. In the first one, we have the activation of the classic cell-proliferation pathways lead by PI3K–AKT–mTOR signaling, MET or IGF signaling cascades and RAS–MAPK cascades. In the second group of HCCs, there is the activation of the non-canonical Wnt signal [23]. On another note, the nonproliferation tumor class is mainly prevalent in alcohol-associated HCC and HCV-related HCC. This group can be characterized by a dominant canonical Wnt signaling pathway linked to mutations in the CTNNB1 gene or by the activation of the IFNα pathway [22,24]. The classification related to immune cell status divides HCC tumors into three groups: immune-active, immune-exhausted and immune-excluded (Table 1). The first two are characterized by immune cell infiltrates of a different nature with the presence, in the context of the tumor, of active helper T (CD4+) and cytotoxic T (CD8+) cell infiltrates. Immune-excluded tumors are characterized by a lack of T cell infiltrates and an enhancement of regulatory T (Treg) cells. They are molecularly dominated by canonical Wnt signaling and additional immune-dissuasive cascades [25]. A third type is defined as immune-exhausted HCC and contains a particular tumor-immune microenvironment (TIME), characterized by T cell exhaustion, the infiltration of the macrophage and fibroblasts and the activation of TGFbeta signaling, together with intra-tumor fibrosis and a high degree of intra-tumor steatosis [26]. This tumor subtype is particularly interesting because it seems highly susceptible to immunotherapy and the degree of steatosis has been considered a histological hallmark of this biological property.

Cholangiocarcinoma (CC) is the second-most common tumor of the liver with a very poor prognosis [27]. It is derived from cholangiocytes, the epithelial cells that line the biliary system. CC is divided into extrahepatic bile ducts, from where two-thirds of CCs originate, and intrahepatic bile ducts, which are the points of origin for the remaining third [28,29]. Due to its poor outcome, it is crucial to understand the mechanism of cholangiocarcinogenesis [30,31]. Several histological and biochemical factors have been hypothesized to be involved in the carcinogenesis and progression of CC, including stem/progenitor cells, tumor microenvironment and genetic and epigenetic alterations, as well as exposure to carcinogenic agents. Hence, numerous classifications have been proposed based on localization, histopathological aspect and molecular alterations [32,33]. One of the most recent classifications divides CCs according to the anatomical localization in intrahepatic (iCC), perihilar (pCC) and distal (dCC) (Table 1). Each of them has a different epidemiology, pathogenesis, and clinical features [28,34]. Moreover, iCC can be subdivided into large and small (or peripheral) CC. The first one originates from large bile ducts with peribiliary glands (PBGs), whereas the second one originates from small ducts in connection with the canals of Hering [35]. In both cases, iCC arises from hepatic stem/progenitor cells, located in the canals of Hering in the PBGs [36]. The histological classification divides iCC into adenocarcinoma with several degrees of differentiation. Both pCC and dCC have the aspect of exophytic or endophytic tumors, and their cells are mucin-producing cholangiocytes [28] (Figure 1B). Recently, an immunohistochemical panel to distinguish and characterize CC was proposed, including markers for intermediate filaments (CK7, CK17, CK19, CK20), CA19–9, markers usually expressed in adenocarcinomas of gastrointestinal and pancreatobiliary origin (mCEA, CA125), mucins (MUC2, MUC5AC) and tumor suppressor proteins (SMAD4) [37]. Furthermore, liver cancer that shows elements of both CC and HCC at the same site is defined as mixed hepatocellular-cholangiocellular carcinoma (cHCC-CC) [38]. They disclose a morphology that can yield a differentiation between mixed hepatocellular-cholangiocellular carcinoma from atypical HCC [39]. In order to understand the molecular mechanism of CC, several studies are under investigation, including an investigation of the Ras-MAPK pathway, which is one of the most critical signals in cholangiocarcinoma pathogenesis [40]. In addition, the transcription factor STAT3 has an important role in promoting tumorigenesis by modulating tumor proliferation and survival [41]. At last, genetic changes are studied. Tumor-suppressive-gene *PTEN* inactivation or loss, together with the activation of AKT or mTOR, were associated with poor patient outcomes in extrahepatic cholangiocarcinoma. In CC, there is an overlap between these genes and those involved in HCC, which are involved in cell-cycle dysregulation, transforming growth factor β (TGFβ)/Wnt pathway activation and increasing α-fetoprotein. All together, these genes are associated with poor outcomes [34,42]. In conclusion, both HCC and CC are heterogeneous tumors with molecular classes, an immune microenvironment and oncogenic drivers different among them. At the moment, it is impossible to find a generic mechanism or a unique therapeutic approach based upon their functional activators; therefore, the management of HCC requires a multidisciplinary approach.

## 3. Iron Metabolism in the Liver

**Figure 2:**
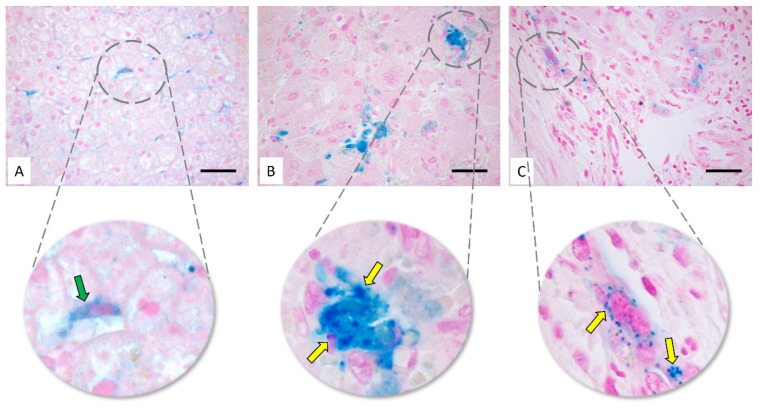
Iron deposition in liver tissues through Perls’ staining in control hepatic tissue (**A**), HCC (**B**) and CC (**C**). The iron deposits are visible as blue areas (green arrow) or blue granules (yellow arrows). Scale bar: 20 μm.

In humans, iron metabolism is a finely regulated process that promotes erythropoiesis, energy balancing of the mitochondria and cell proliferation. Iron is a chemical element well known for its role as a constituent of hemoglobin; therefore, it is essential for the transport of oxygen to tissues. In addition, iron is also involved in the following: (i) transforming ribonucleotides to deoxyribonucleotides during DNA synthesis; (ii) the mitochondria electron transport chain; and (iii) muscle physiology via myoglobin synthesis [43]. Despite its physiological importance, free circulating iron is toxic to tissues and damages the biological membranes and DNA through Fenton’s reaction. Particularly, Fe^2+^ reacts with hydrogen peroxide (H_2_O_2_) to generate Fe^3+^ ions and highly reactive and harmful free hydroxyl radicals (OH) [44]. As a consequence, the organisms have developed sophisticated control mechanisms for the maintenance of iron homeostasis to regulate intra- and extracellular traffic and tissue storage. Alterations of this homeostasis can cause well-known disorders, such as hereditary hemochromatosis, iron-deficiency anemia, sideroblastic anemia, spherocytosis or iron-overload diseases, which occurs frequently in polytransfused patients [45]. Because the liver is the main organ of iron storage, it is one of the first targets of iron-overload toxicity [46]. A 70 kg man has a total amount of iron ranging from 4 to 5 mg. Most of it is bound to hemoglobin, 10% is bound to muscle myoglobin and 20–30% is found in the liver, spleen and bone marrow bound to both ferritin and hemosiderin. In liver, iron is approximately 0.4 mg total, and it is mainly localized in hepatocytes bound to ferritin, hemosiderin, heme and low-molecular-weight intracellular compounds, which act as intermediates for a wide variety of biochemical processes [47]. Histologically, ferric iron can be observed under an optical microscope through a Perls stain that colors the Fe^3+^ ion in Prussian blue and demonstrates its accumulation in some pathological conditions [48] (Figure 2). Hepatocytes are the main cells responsible for the metabolism and storage of iron because they have a high synthesis capacity of ferritin, but also of ceruloplasmin, hemopexin and haptoglobin [49]. Under physiological conditions, the liver imports iron, mainly carried by transferrin. In some pathological conditions in which iron is in excess, non-transferrin-bound iron (NTBI) also contributes to hepatic uptake. NTBI has been found to be higher in the plasma of patients with pathological iron-overload conditions in which transferrin was fully saturated [50]. The main role of the liver as a regulatory center for iron became evident after the discovery of a small peptide (25 aa) called hepcidin with antimicrobial and antifungal properties. Hepcidin is encoded by the *HAMP* gene and secreted by hepatocytes as a hormone-like phenotype [51,52]. It regulates iron flux by modulating Ferroportin, a ionic iron transporter expressed on cell membranes [53], particularly in tissues and cells associated with iron transport, such as duodenal enterocytes, Kupffer cells, splenic red pulp macrophages, periportal hepatocytes and placental syncytiotrophoblast [54]. Ferroportin regulates the transport of iron across the cell membrane and its delivery outside the cell where it can be bioavailable for tissue demands. Hepcidin can bind to the extracellular domain of Ferroportin to inhibit its functions and to limit iron absorption and mobilization across the plasma membrane of enterocytes and hepatocytes, thus reducing its plasma concentration in order to maintain homeostasis [53]. Iron, inflammatory and erythropoietic signaling can modulate the production and transcription of the HAMP gene. The increase in plasma and tissue iron levels also promotes the BMP/SMAD (bone morphogenetic protein/suppressor of mothers against decapentaplegic) activation pathway through transferrin receptors (TfR1 and TfR2) and the HFE (homeostatic iron regulator). The activation of this last pathway induces the formation of a SMAD1/5/8-phosphorylated transcription complex linked to SMAD4, which binds the HAMP promoter and induces the transcription of *HAMP* gene. Other evidence suggests that the *HAMP* gene can be transcribed directly from the HFE protein via the Erk1/2 pathway [55]. Another activation pathway is mediated by inflammatory stimuli, such as IL-6 and its receptor, expressed on hepatocytes that induce the activation of the JAK/STAT3 pathway and the transcription of the HAMP gene [56]. Another regulatory pathway is inhibitory in hepcidin synthesis. This is linked to the synthesis of erythroblast-derived hormone erythroferrone (ERFE). The production of erythropoietin by the kidney, the sensor of plasmatic oxygen tension, induces the synthesis of erythroblast in bone marrow [57]. A high ERFE protein concentration induces the proliferation of the erythroblast in bone marrow in the case of reduced oxygen tension. The increased number of erythroblasts increases the circulating ERFE. The increased ERFE induces, in turn, a reduction in hepcidin gene transcription within the liver through the inhibition of BMP6/SMAD [58]. This mechanism allows more iron to pass towards the cell membranes to be internalized by other cells, used and fluxed out. There are at least four ways of internalizing iron at the cellular level. The most important pathway is mediated by transferrin, a plasma protein that binds and carries two ferric ions. Iron internalization is regulated by the membrane transferrin receptor (TfR1), which binds transferrin but not apotransferrin (free transferrin not bound to iron). The transferrin receptor (TfR1) mediates cellular iron uptake through the clathrin-dependent endocytosis of iron-loaded transferrin [59]. After endocytosis, endosomal acidification by proton entry triggers a conformational change in TfR1 and Tf that causes a release of iron into the cytoplasm [60]. Another iron-internalization process is through the NTBI family, a class of transporters that includes both the DMT1 (divalent metal transporter 1) and the ZIP14 and ZIP8 proteins [61]. ZIP8 and ZIP14 have been described as crucial in the transport of iron Fe^3+^ by reducing Fe^2+^ by the prion protein (PrP^c^), which acts as a ferrireductase and promotes iron internalization [62,63]. PrP^C^ knockout mice are in fact subject to iron deficiency and, on the contrary, iron overload occurs in the brains of patients affected by prion disease and contributes to neuronal death [64,65]. A third mechanism is based on the internalization of iron bound to hemoglobin through CD163, a scavenger receptor expressed exclusively in the cells of the monocyte–macrophages system, including the Kupffer cell of the liver. The upregulation of CD163 is the main element able to switch macrophages to an activated phenotype. It is therefore important in the pathogenesis of hepatitis and cirrhosis after hemochromatosis. Furthermore, the scavenging of hemoglobin by the CD163 receptor is critical during physiological or pathological hemolysis to circumvent hemoglobin (Hb)/heme-induced toxicity [66]. In such conditions, CD163 mediates hemoglobin endocytosis, the formation of the haptoglobin-hemoglobin complexes and the transformation of the heme group into biliverdin through the action of heme oxygenase (HO-1) in the lysosomes [67]. A fourth mechanism of iron internalization is based on the action of SCARA5 receptors (SCAvenger Receptor class A member 5) that bind the iron carried by ferritin and drive it towards the lysosomes to be subsequently reduced, released and used as Fe^2+^ by the cell [68]. As previously described, Fe^2+^ is finally exported outside the cell thanks to Ferroportin, but it is immediately oxidized to Fe^3+^ by ceruloplasmin or hephaestin and is bound and transported again by transferrin [69].

## 4. Ferroptosis in Hepatocarcinoma

As previously described, ferroptosis, a new form of RCD, is linked to iron overload and oxidative-stress-induced lipid peroxidation [8]. It is morphologically characterized by a loss of plasma membrane integrity, its disruption with the consequent release of intracellular components and the typical hyperpolarization of the mitochondrial membrane [70,71]. Inducible cell death represents a common approach in tumors to contrast the progression of the disease. Recently, ferroptosis, has been introduced as a possible therapeutic strategy against several liver tumors, including HCC [72,73]. Indeed, tumor cells show a higher request of iron with a consequent increase in oxidative stress levels. To prevent cell death, tumor cells try to block oxidative stress by activating antioxidant genes or molecular factors involved in the regulation of the ferroptosis. Some of them are GPX4 and SLC11A2 (soluble carrier protein 11A2), which are upregulated in HCC compared to the healthy liver [74,75]. In order to support the ferroptotic process, with the aim of reducing tumor growth, a first possible strategy would be to prevent the activation of the antioxidant protection system by the tumor cells and to maintain high levels of iron in the cell [76]. Initial preclinical data regarding the induction of ferroptosis in HCC have shown promising results in contrasting cancer growth [10,77]. The main determinants of ferroptosis are iron overload and lipid peroxidation. In order to contrast HCC, one strategy might be to increase abnormal iron levels, leading to a promotion of an overproduction of ROS and inducing cell death. Artesunate (ART) is a drug commonly used for malaria, but it can induce ROS-dependent ferroptosis with damage of the endoplasmic reticulum (ER) and consequent cellular damage [78]. Additionally, divalent metal-ion transporter-1 (DMT1), which is a transmembrane iron transporter expressed by hepatocytes and enterocytes, has been associated with increased mitochondrial oxidative phosphorylation and glycolysis, affecting both mitochondrial function and iron homeostasis [79]. On the other hand, sorafenib (SOR) is a multi-tyrosine kinase inhibitor used for the treatment of HCC, as well as kidney and thyroid carcinomas. Pharmacologically, it is able to inhibit angiogenesis by acting on the vascular endothelial growth factor receptor (VEGFR) and platelet-derived growth factor receptor (PDGFR) [80,81]. Interestingly, ferroptosis is closely associated with the presence of aerobic metabolism, which is able to maintain continuous lipid peroxidation. This is also proved by reperfusion injury diseases, where increased blood flow to a damaged tissue induces a cascade of events, including oxidative stress, ROS production and cell death [82]. In this view, the inhibition of angiogenesis would be counterintuitive in the induction of ferroptosis as possible tumor arresting mechanism. However, hypoxia and hypo-vascularization themselves increase the production of ROS, triggering a compensatory loop [83]. For this reason, a combined approach, with an initial induction of ferroptosis by cellular iron overload and the subsequent inhibition of the blood supply, would be a possible integrated strategy to maximize tumor arresting in HCC. In this way, in fact, ferroptosis reduces tumor growth within a highly vascular and oxygenated environment. Subsequently, the reduced blood supply induced by VEGF inhibitors triggers a further increase in ROS and avascular necrosis of the tumor [84]. Another approach might be related to polyunsaturated fatty acids (PUFAs) that enhance the susceptibility of HCC cells to ferroptotic cell death. Indeed, PUFAs can inhibit liver inflammation and decrease the formation of some tumorigenic factors, such as cyclooxygenase-2 (COX-2), β-catenin and tumor necrosis factor-α (TNF-α), leading to a reduction in HCC proliferation [85,86]. It has been also demonstrated that eating foods rich in saturated fatty acids decreases the probability of developing HCC, and it is very important to understand how changes in PUFAs, iron homeostasis and monounsaturated fatty acids can also mediate the immune control of HCC [87]. An interesting point is also the pre-treatment morphological analysis of the tumor. Because unsaturated fatty acids are involved in the peroxidation mechanism of ferroptosis, a tumor characterized by steatosis would probably be more sensible to therapeutic approaches targeting ferroptosis. For this reason, exhausted-type HCC, which is characterized by a high degree of intra-tumor fibrosis and steatosis [26], is a possible ideal candidate for therapeutic approaches based on the induction of ferroptosis. Sorafenib (SOR) is considered fundamental for its function in inhibiting soluble epoxide hydrolase (SEH). In particular, SEH induces the conversion of arachidonic acid (AA) and omega-3 docosahexaenoic acid (DHA) in the corresponding glycols, which might support tumor progression and metastasis, whereas DHA 19,20-epoxydocosapentaenoic acid (19,20-EPD) has antagonist effects. Therefore, treatment with SOR and DHA may reduce tumor growth in HCC [88]. A recent study suggested that oncoprotein hepatitis B X-interacting protein (HBXIP) plays a role in preventing SOR-induced ferroptosis in HCC cells. In fact, SOR downregulates HBXIP expression, increasing malondialdehyde (MDA) production and glutathione (GSH) depletion and therefore supporting SOR-mediated ferroptotic cell death. In detail, HBXIP leads to the expression of stearoyl-CoA desaturase (SCD), and the subsequent activation of the HBXIP/SCD pathway decreases the anticancer activity of SOR [89]. Although SOR is a key molecule in the treatment of HCC, by acting on several mechanisms involved in tumor growth, including ferroptosis, there are some biological and clinical factors in HCC that can enhance or inhibit the SOR effect that need to be considered before deciding upon this therapeutic approach. The overexpression of the cysteine-rich secretory acidic protein (SPARC) determines oxidative stress, inducing ferroptosis and promoting the release of lactate dehydrogenase (LDH). It blocks the expression of proteins preventing ferroptosis and increases the toxic effects of SOR [90]. Glutathione s-transferase (GSTZ1), an enzyme linked to phenylalanine metabolism, is downregulated in SOR-resistant HCC cells. Lastly, QSOX1 is a factor able to prevent EGF-induced EGFR activation by promoting ubiquitin-mediated EGFR degradation and reducing NRF2 (nuclear factor E2-related factor 2) activity, which linked to the reduction in ROS and the defense against oxidative stress, supporting SOR-induced ferroptosis by inhibiting NRF2 [91]. Furthermore, from a clinical point of view, the use of SOR is still correlated to several side effects, which also need to be considered and evaluated on a patient-by-patient basis. Ferroptosis is a fundamental form of regulatory cell death and the use of ferroptosis inducers provides new possibilities for the treatment of HCC [92]. For this propose, the molecular and morphological analysis of the tumor should be the first step in establishing whether ferroptosis represents a good therapeutic target for a specific patient. The modulation of ferroptosis, in combination with the modern molecular diagnostic and drug delivery, will contribute to improvements in the prognosis and therapy of HCC.

## 5. Ferroptosis in Cholangiocarcinoma

In recent years, several possible strategies have been studied to contrast malignant CC, together with the evaluation of possible new targets for diagnosis and treatments. Interestingly, one of the mechanisms involved in the resistance of tumors to different therapies is dependent on the lipid peroxidase pathway. For this reason, ferroptosis may be considered a new method to overcome this resistance, as already reported in ovarian cancer and colorectal cancer [93,94,95]. The obstructive jaundice of CC determines the direct contact of bile fluid with tumor cells and the possible concentration of biomarkers inside it. Iron metabolism in bile has been evaluated as dysregulated in CC [96]. One of the most important activities of bile acid is to control cysteine catabolism and regulate hepatic sensitivity to oxidative injury. As a consequence, defects in bile-acid homeostasis during pathophysiological conditions damage the antioxidant defense mechanism, leading to an enhancement in oxidative stress [97]. Indeed, ferroptosis might start with the depletion of cysteine, usually found in metal-binding sites, or the inhibition of phospholipid glutathione peroxidase (GPx), a key upstream regulator of the process [3]. Changes in GPx are correlated with tumor development through the accumulation of lipid-derived ROS and consequent ferroptotic death [98]. In particular, the depletion of GPx and the inadequate cysteine input determine the reduction in ferrous iron [Fe^2+^], depletion of GSH level and, more likely, tumor progression [99]. Moreover, many transcription factors involved in oncogenic pathways, such as P53 and myc, are enriched in tissues during CC, while ROS and VEGFR are involved in the induction of transcription factor activity [100]. Therefore, it is very important to analyze bile and its components, which can become biomarkers for early diagnosis, and they are fundamental in revealing the role of iron-induced ferroptosis in CC and, therefore, in establishing whether a therapeutic approach aimed at inducing ferroptosis would be effective. Photodynamic therapy (PDT) represents an innovative method for tumor treatment through the production of ROS without cross-resistance to chemotherapy, and it is recognized as an emerging palliative treatment in unresectable extrahepatic CC [101]. Furthermore, the hydroporphyrin photosensitizer Chlorin A (Chlorin A), which is part of the class of heterocyclic tetrapyrroles derived from porphyrins, is mainly located at the level of the mitochondria, which the main site of ROS production during PDT, but it is also expressed in the lysosomes and in the endoplasmic reticulum, indicating additional mechanisms of Chlorin-A-mediated PDT [102]. Recent studies support the concept that the photosensitizer Chlorin e6 and its derivate Chlorin A play a role against CC. Chlorin A is more powerful than temoporfin, a photosensitizer frequently used against some types of tumors, including CC, because it increases the rate of autophagy and apoptosis in human CC cell lines. Chlorin A-PDT enhances the levels of the phagosome-membrane elongating protein LC3-II, the autophagosomal protein Beclin 1 and the autophagy-mediating pathway PI3K/AKT/mTOR. Moreover, Chlorin A-PDT induces the apoptosis of CC cells originating from the autophagic process [103,104]. This intrinsic apoptotic process is linked to mitochondrial dysfunction with an upregulation of cytochrome c and cleaved caspase 3 levels. Mitochondrial depolarization releases cytochrome c and activates caspase 3, inducing the intrinsic apoptotic pathway [105]. Although, Chlorin A-PDT can inhibit CC growth by increasing apoptotic cells in tumor tissues, other studies have reported prosurvival effects of the photosensitizer Chlorin e6-PDT [106,107]. For this reason, and because the role of PDT in CC is still unclear, it is necessary to study potential factors involved in PDT-mediated ferroptosis to better approach this emerging therapy and find possible biomarker candidates to stratify the therapeutic approach [99]. For this propose, several molecules are worthy of attention. Solute carrier family 7 member 5 (SLC7A5) belongs to the glucose transporter family and plays a crucial role in the composition of amino acid transporters during the movements of cystine and other amino acids by controlling autophagy, glutathione synthesis and glutamine decomposition [108]. SLC7A5, known as LAT1 (large amino acid transporter 1), is upregulated in different malignant tumors, and it is linked to severe outcomes in CC [109]. Additionally, ZEB1, a transcription factor, is an epithelial–mesenchymal transition regulatory that mediates the transition from epithelial to mesenchymal cells in CC [110]. Recent data have reported that ZEB1 is significantly upregulated after PDT, whereas SLC7A5 is downregulated [111]. The direct stimulation with PDT can block the mesenchymal phenotype in tumor cells and the use of erastin may inhibit the SLC7A11-enhancing effect of PDT [112]. According to these results, SLC7A5 could be considered a resistance factor in PDT-induced ferroptosis. The activation of several signaling pathways is related to a poor prognosis of CC, such as the phosphatidylinositol signaling system and the PI3K/AKT/mTOR pathway. They are responsible for extracellular signaling factors that induce the release of secondary messengers, such as inositol 1,4,5-trisphosphate (IP3) and diacylglycerol (DG) [113,114]. At last, the activation of macrophage Fcγ receptors (FCγRs) is linked to a short survival in CC patients. It regulates phagocytosis and the acquisition of lysosomal proteases, together with scavenging from ROS products [111]. Taking into consideration both the release of ROS and the fusion with lysosomes, FCγR-mediated phagocytosis can regulate the balance between macroautophagy and ferroptosis. All together, these findings suggest that treatments that involve blocking or activating these survival-related pathways could play a role in the management of CC patients. The potential effect of PDT to induce ferroptosis has been explored, not only in CC cell lines, but also in organoid models. Cell viability and colony-formation experiments have showed the inhibitory role of PDT in organoids, whereas experiments dedicated to the study of oxidative stress have shown, after sinoporphyrin sodium (DVDMS) and PDT treatment, an increase in ROS production, leading to an enhancement of MDA and an impairment of GSH in CC [111]. In conclusion, cholangiocarcinoma is an extensively heterogeneous tumor, which shows large differences in patient etiology and in the variety of pathways and gene functions. In such a context, the role of ferroptosis might be related to the upstream pathways of endoplasmic reticulum stress, autophagy and apoptosis. The identification of ferroptosis-related genes seems to correlate well with the prognosis of CC, thus representing a predictive signature of survival, susceptibility to photodynamic therapies and treatments, while also confirming the central role of ferroptosis in liver disease [111,115]. Therefore, the activation or inhibition of these pathways can be a relevant contributor to the overall survival of patients.

## Figures and Tables

**Table 1:** Schematic classification of hepatocellular carcinoma and cholangiocarcinoma.

HCC		CC	
**molecular subtypes:**		**anatomical location:**	
Proliferation class	➢ proliferation progenitor cell type	Intrahepatic (iCC)	➢ large
	➢ proliferation-Wnt-TGFβ type		➢ small or peripheral
Non proliferation class		Perihilar (pCC)	
		Distal (dCC)	
**based on cell origin:**		**gross classification of iCC:**	
Transformed mature hepatocytes		Mass-forming type	
Liver stem cells		Periductal infiltration	
		Intraductal growth type	
**related to the immune cell status:**		**histology of iCC:**	
Immune active		Involving the large bile ducts	
Immune-exhausted		Involving the smaller ducts	
Immune-excluded			
**Mixed hepatocellular-cholangiocellular carcinoma (cHCC-CC)**			

## Data Availability

Not applicable.
